# Social support, disclosure and stigma and the association with non-adherence in the six months after antiretroviral therapy initiation among a cohort of HIV-positive adults in rural KwaZulu-Natal, South Africa^*^

**DOI:** 10.1080/09540121.2018.1549720

**Published:** 2018-11-25

**Authors:** S. George, N. McGrath

**Affiliations:** a Faculty of Medicine, University of Southampton, Southampton General Hospital, Southampton, UK; b Academic Unit of Primary Care and Population Sciences and Department of Social Statistics and Demography, University of Southampton, Southampton, UK; c School of Nursing & Public Health, Africa Health Research Institute, University of KwaZulu-Natal, KwaZulu-Natal, South Africa; d Research Department of Epidemiology & Public Health, University College London, London, UK

**Keywords:** HIV, antiretroviral therapy, non-adherence, South Africa, social support

## Abstract

The World Health Organisation (WHO) recommends antiretroviral treatment (ART) initiation at human immunodeficiency virus (HIV) diagnosis. As ART programmes expand, addressing barriers to adherence is vital. Past mixed findings on the association between social support, stigma and non-disclosure with ART adherence highlights the need for further research. The primary aim of this study was to examine how these factors are associated with ART non-adherence in the six months after ART initiation. The secondary aim was to explore how other factors are associated with non-adherence. We conducted secondary analysis of prospective data from HIV-positive adults initiating ART. Social support, disclosure patterns, perceived stigma and other demographic factors were collected at ART initiation and six months follow-up. Logistic regression models were used to examine factors associated with self-reported ART non-adherence in the last six months and the last month before the six month follow-up (“recent”). Non-adherence in the last six months was twenty-five percent and recent non-adherence was nine percent. There was no association between non-adherence and social support, stigma or non-disclosure of HIV status. In the final model the odds of non-adherence in the last six months were significantly higher for those: with incomplete ART knowledge (aOR 2.10, 95%CI 1.21–3.66); who visited a healthcare provider for conditions other than HIV (aOR1.98, 95%CI 1.14–3.43); had higher CD4 counts at ART initiation (CD4 100–199:aOR 2.50, 95%CI 1.30–4.81; CD4 ≥ 200:aOR 2.85, 95%CI 1.10–7.40;referent CD4 < 100 cells/mm^3^); had tested HIV-positive in the last year (aOR 2.00, 95%CI 1.10–3.72; referent testing HIV-positive outside the last year); experienced a rash/itching secondary to ART (aOR 2.48, 95%CI 1.37–4.52); and significantly lower for those ≥48 years (aOR 0.65, 95%CI 0.46–0.90). Early non-adherence remains a concern. Incorporation of adherence monitoring and ART knowledge enhancement into appointments for ART collection may be beneficial.

## Background

South Africa (SA) has the most cases of HIV globally with an estimated 7.1 million positive individuals and the largest antiretroviral treatment (ART) programme worldwide (Africa, 2014; Motsoaledi, 2014). The WHO recommends ART initiation following HIV diagnosis regardless of CD4 count. Strict adherence to ART is required to prevent treatment failure, drug resistance and reduce transmission (Department of Health, 2016; Peltzer, 2012; Reid, 2016). For those with treatment failure in low and middle-income countries few alternatives to first-line drug regimens are available, making adherence vital (National Department of Health, 2015). HIV is now a chronic disease. Chronic disease literature based in sub-Saharan Africa (SSA) has documented poor adherence and the importance of social support in conditions which require life-long treatment (Adegbola, Marincowitz, Govender, & Ogunbanjo, 2016; Loeliger, Niccolai, Mtungwa, Moll, & Shenoi, 2016; Mendenhall & Norris, 2015; Ncama et al., 2008; Oni et al., 2014; Osamor, 2015). However, high levels of HIV-related perceived stigma can inhibit HIV disclosure and thus prevent patients from accessing social support (Bhengu et al., 2011; Hunter-Adams et al., 2017; Ncama et al., 2008; Osamor, 2015; Treffry-Goatley et al., 2016). The closely interwoven and likely dynamic nature of disclosure, stigma and social support make this a challenging research area (Treffry-Goatley et al., 2016). A meta-analysis, incorporating studies from 2006 to 2016 of HIV-positive adults taking ART in SSA found greater perceived stigma was associated with greater odds of non-adherence, while higher levels of social support and disclosure of HIV status to friends and family were facilitators of adherence (Croome, Ahluwalia, Hughes, & Abas, 2017). A US study looking at the psychosocial factors and ART adherence in HIV-positive adults found that social support acted as a modulating factor for the association between adherence and self-efficacy and stigma rather than being directly associated with non-adherence (Diiorio et al., 2009; Simoni, Frick, & Huang, 2006). A similar study in KwaZulu-Natal (KZN), SA found an association between non-adherence and perceived stigma related to HIV disclosure but not social support (Ncama et al., 2008). Studies in SA suggest that although disclosure can lead to support from a social network there are high levels of perceived stigma with initial disclosure often to a trusted family member rather than a partner (Maman et al., 2003; Maman, van Rooyen, & Groves, 2014). These differences suggest that the association between adherence, social support, disclosure and stigma is complex and dynamic.

Research has identified other factors associated with non-adherence in SSA. Drug and alcohol use, increased health seeking behaviour, the presence of side effects, low socioeconomic status, male gender, poor ART knowledge and less equitable gender norms have been found to be associated with increased non-adherence in SSA (Morojele, 2014; Nyamhanga, Muhondwa, & Shayo, 2013; Peltzer et al., 2011; Peltzer & Ramlagan, 2011; Peltzer, Friend-du Preez, Ramlagan, & Anderson, 2010).

The primary aim of this study was to contribute to the literature by examining how social support, perceived stigma and disclosure of HIV status and ART initiation are associated with non-adherence using prospective data from a cohort study conducted in rural KZN, South Africa from 2009 to 2013 among HIV-positive adults initiating ART. The secondary aim was to explore how previously identified factors are associated with non-adherence in KZN.

### Methodology

Details of the study design and cohort at enrolment have been described elsewhere (McGrath, Richter, & Newell, 2011, 2013). Men and women accessing the HIV treatment and care programme in local primary care clinics, aged 18 years or older, were screened for study eligibility when attending clinic for their CD4 test result. Individuals who were eligible for ART according to national guidelines in 2009 (with CD4 < 200 cells/μl or WHO Stage IV HIV disease) and those with CD4 > 500 cells/μl were eligible to enrol in the cohort study. A cut off from CD4 > 500 for enrolment was chosen as these individuals have been shown to take on average 2.5 years to progress to CD4 < 200. This ensured time for repeated questionnaires before initiating ART allowing comparison with those on ART*.* A questionnaire administered at enrolment collected perceived HIV stigma, social support, HIV testing history and reports of disclosure of HIV status and ART initiation as well as other socio-demographic variables (Table 1). This study focused on the 385 adults who were eligible for ART at enrolment and uses data from enrolment and the first follow-up visit at six months. At six months, a questionnaire was administered to explore whether the individual’s circumstances had changed since enrolment. Information was also collected for alcohol use, health seeking behaviour, disclosure of ART initiation, ART regimen, ART adherence and side effects.

**Table 1.** Data collection measures for social support, disclosure, ART side effects and health seeking behaviour variables.

Questions used to measure social support^a&b^	Possible answers	Grouping for analysis
How often do you spend time with family?	Every day, several days a week, at least once a fortnight, once a month, less than once a month.	No change in the groupings for analysis
How often do you spend time with friends?	Every day, several days a week, at least once a fortnight, once a month, less than once a month.	For analysis answers were grouped as: Every day/several days a week At least once a fortnight Once a month/Less than once a month
How much can you rely on family/friends if you have a serious problem?	A lot, a little, not at all.	No change in the groupings for analysis
How much can you open up to family/friends to talk about worries?	A lot, a little, not at all.	No change in the groupings for analysis
Do you have someone in your life to tell your private feelings and your concerns	Yes, No.	No change in the groupings for analysis
Disclosure Questions	Possible Answers	Grouping for analysis
Who have you disclosed your HIV status to? Have you disclosed to anyone that you have started ART, and if yes, to whom.	For both questions if yes, individuals disclosed to were chosen from a pre-written list: partner. Children, mother, father, sister, brother, other female relative, other male relative, female friend, male friend, doctor/nurse, traditional healer, priest/church elder, HIV support group, other	For analysis answers were grouped as: Not disclosed Disclosed to family Disclosed to friends Disclosed to both family and friends
Symptom self-report	Possible Answers	Grouping for analysis
Have you experienced symptoms over the last six months which you think may be due to your ART?	If yes, symptoms experienced were recorded from a pre-written list: diarrhoea, nausea, rash/itching, pain in hands/feet, feeling tired, insomnia headaches, body shape changes, and a category ‘other’ allowed for other symptoms to be specified.	We counted the total number of symptoms each individual reported and then grouped this as: Zero symptoms One symptom Two symptoms Three or more symptoms Symptoms were also considered individually as binary variables in models that did not consider the number of symptoms
Health-seeking Behavior	Possible Answers	Grouping for analysis
Have you visited any of the following to treat diseases or conditions other than HIV/AIDS?	Responses were chosen from: this clinic, another government clinic, hlabisa: hospital, another government hospital, private clinic or hospital, inyanga, sangoma, umthandazi, pharmacy or other.	Responses were grouped into public healthcare facilities (Government clinics, government hospitals and Public Health Care Hospital), separate from traditional healer and considered as two separate variables in the analysis.

^a^Questions related to social support and HIV disclosure were asked at enrolment – these were used to ensure that we had temporally the correct value of the variable before any “non-adherence” took place. All other questions were asked at the six month visit.

^b^Questions regarding social support were derived from Myer *et al* (Myer, Stein, Grimsrud, Seedat, & Williams, 2008).

A 28-item scale was used to measure perceived HIV stigma. The scale was designed to take into account themes related to ART use and the healthcare setting (Sayles et al., 2008). Social support was assessed using five questions. HIV disclosure was assessed at baseline and ART initiation disclosure assessed at six months (Table 1). Participants were asked at the six month visit about their health-seeking behaviour (visiting a healthcare provider) for anything other than HIV in the past six months and if they had experienced side-effects from their ART (see Table 1). They were also asked: “*When was the last time that you missed taking your antiretroviral pills?*” The answer options were; “*missed more than a month ago*,” “*less than a month ago,*” “*last week*,” “*earlier this week*,” “*yesterday*” and “*I have never missed*.” Scales used to measure participants’ views on gender norms and ART knowledge have been described in detail elsewhere (McGrath et al., 2011; Pulerwitz & Barker, 2008; Sayles et al., 2008).

This paper was developed as a year-long research project for the first author as a University of Southampton medical student. Ethics approval for the analyses for this paper was given by the University of Southampton Human Research Ethics Board in line with their undergraduate research policy. The study that provided the secondary data had ethics approval for data collection and analysis from the University of KZN (ref BF083/08) and the London School of Tropical Medicine and Hygiene (ref 5413), and permission to conduct the study in government clinics from the Provincial Department of Health in KZN.

## Outcomes

Non-adherence for the primary analysis was defined as a binary variable representing having missed one or more doses over the last six months with a value of one assigned for those who had missed doses versus zero for those who answered, “I have never missed.” A second outcome, recent non-adherence, was defined as having missed one or more doses in the month before the six month interview.

## Analysis

Individuals eligible for ART at enrolment who had initiated ART for six months and were interviewed at six months were included in this analysis. Characteristics at enrolment of those eligible and not-eligible for the analysis were compared to explore differences between groups and whether the analysed sample broadly represented all those enrolled. Non-eligible individuals were excluded from further analysis.

**Figure 1.**
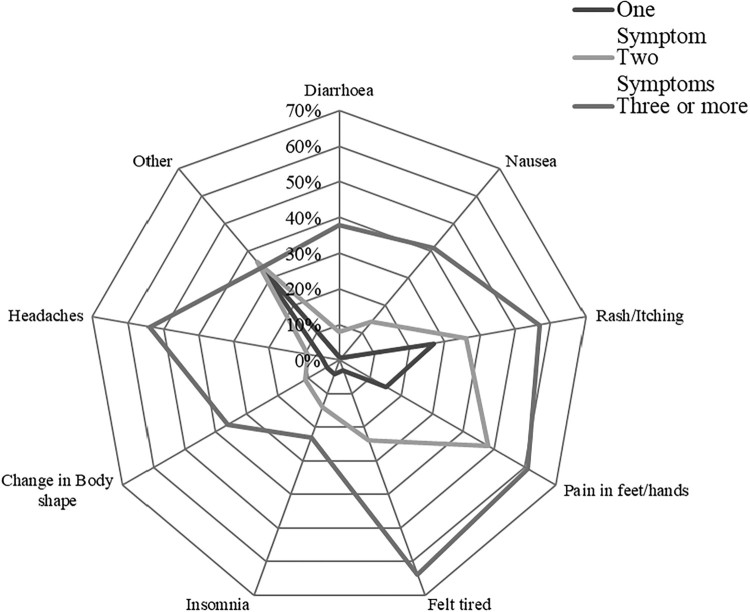
Radar chart for ART Side effects by six months. Note: Of the seventy-seven participants who described experiencing “other” side effects: 82% reported dizziness. The others were: shortness of breath (2) and bad dreams (2), acid, back pain, bleeding ears, hair loss, no period, tremor, feeling cold, sight problems, loss of appetite, mouth sores, sores on their head.

We used STATA SE 14 for all analyses. Descriptive analyses used t-tests for continuous variables, Wilcoxon rank sum tests for skewed distributions, and chi-square tests for categorical variables. A radar chart was used to present the prevalence and type of symptom reported in response to the symptom self-report question (Figure 1) and for those reporting multiple symptoms. For both non-adherence outcomes (non-adherence in the last six months and recent non-adherence), we conducted univariable logistic regression and all variables with a likelihood ratio test *p*-value <0.1 were considered for the multivariable models. We used forward and backward stepwise regression to build the final model for each outcome which was the most parsimonious model possible.

In initial regression models, age at enrolment was considered a categorical variable with four categories (18–27, 28–37, 38–47 and ≥ 48 years) in line with a similar study (Bhat et al., 2010). However, estimates indicated that age could be represented by a binary indicator (below 48 years/48 years and above) in the models. Stigma was represented as a binary indicator distinguishing the quartile of individuals with the greatest perceived stigma from all other individuals. ART knowledge at time of enrolment was considered in the models. Disclosure of ART initiation to a partner was also considered univariably.

For our second adherence outcome definition, we applied the final model for non-adherence in the last six months to the recent non-adherence outcome. Additionally, we conducted an independent model-building exercise that looked at factors associated with recent non-adherence.

### Results

## Sample

Of the 385 in the ART group, 321 (83%) had initiated ART by six months and were eligible for analysis (Table 2). Characteristics of those included in the analyses were similar to the entire sample enrolled, except that a smaller proportion of men were included (*p* = 0.02). Of the 64 not included (32 males), 33 had died (15 males), two had relocated (males), seven refused the follow up questionnaire (five males) 19 were lost to follow up (nine males) and one had missing data. Among those included in the analysis, 66% were female. Sixty-four percent currently had one or more sexual partners and 56% were directly in receipt of a government grant*.* Eighty-one percent had self-initiated the HIV test that diagnosed their HIV-positive status, 60% due to sickness. Fifty-six percent reported spending time with their family every day but only 18% reported seeing their friends every day. Twenty-nine percent reported high levels of perceived stigma.

**Table 2.** Demographics and HIV characteristics at enrolment by eligibility for analysis.

Characteristics	Eligible for analysis (*N *= 321) n (% of N)	Not eligible for analysis (*N *= 64) n (% of N)	Chi squared *p* value
*Sex*			
Female	212 (66)	32 (50)	0.02
Male	109 (34)	32 (50)	
*Current Marital Status*			
Never Married	247 (77)	57 (89)	0.23^a^
Currently Married	45 (14)	4 (6)	
Separated/Widowed	29 (9)	3 (5)	
Age: *Median (IQR)*	*36 (30–44)*	*33 (28–41)*	0.13^b^
< 48	273 (85)	54 (84)	0.89
≥ 48	48 (15)	10 (16)	
*Education (Missing, N = 26)*			
<1yr	19 (6)	5 (10)	<0.66^a,c^
Primary School	76 (25)	10 (20)	
Secondary not matric	128 (41)	21 (41)	
Matric & higher	85 (27)	15 (29)	
Socioeconomic status			
Current Employment:			
Yes	84 (26)	13 (20)	0.32
No	237 (74)	51 (80)	
*Perceived Stigma:*	* *	* *	* *
Lower perceived stigma	229 (71)	48 (75)	0.55
Higher perceived stigma	92 (29)	16 (25)	
*Disclosed HIV + at enrolment:*			
Not Disclosed	35 (11)	12 (19)	0.14^a^
Disclosed to FAM	241 (75)	40 (63)	
Disclosed to FRI	3 (1)	0 (0)	
Disclosed to both	42 (13)	12 (19)	
*Has someone to confide in*			
Yes	301 (95)	59 (92)	0.38
No	16 (5)	5 (8)	
*Can rely on family and friends*			
A lot	244 (76)	52 (81)	0.58
A little	66 (21)	11 (17)	
Not at all	11 (3)	1 (2)	
*Can be open with family and friends*			
A lot	122 (38)	24 (38)	
A little	119 (37)	24 (38)	0.99
Not at all	80 (25)	16 (25)	
*ARV Knowledge at enrolment*			
100% correct answers	157 (49)	30 (46)	0.77
< 100% correct answers	164 (51)	34 (53)	
*CD4 Categories*^d^			
0–99 cells/ µl	103 (32)	24 (39)	0.46^a^
100 – 199 cells/ µl	183 (57)	33 (54)	
≥200 cells/ µl	35 (11)	4 (7)	
*1st line ART Drug Regime (missing, N = 3)*			
AZT + 3TC +EFV	2 (1)		
TDF + 3TC + EFV	11 (3)		
D4T +3TC + EFV	253 (80)		
D4T +3TC + NVP	52 (16)		
*Tested Positive <1 year before the study*			
Yes	212 (66)	44 (69)	0.68
No	109 (34)	20 (31)	
*Reported side effects from ART*			
None	100 (31)		
1	100 (31)		
2	67 (21)		
>2	54 (17)		

^a^Fishers Exact Test.

^b^Wilcoxon rank sum test

^c^The statistical test compared the non-missing data. Significant results in bold.

^d^Only 36% had a CD4 measurement taken between their enrolment and the six month visit, thus CD4 at enrolment was considered in the analysis as a proxy for health status at enrolment and represented by three categories: 0–99 cells/µl, 100–199 cells/µl and ≥ 200 cells/µl.

ART was started after enrolment, median 16 days (IQR 7, 28). Ninety-three percent had disclosed they were taking ART to at least one individual at the six month visit. Of those who had disclosed to family members that they were taking ART, 62% of females had told their partner compared to 83% of males (*p *=< 0.001). Ninety-one percent of those who had told a family member did so within one month of ART initiation. Sixty-seven percent had disclosed that they were taking ART to more than one family member. Thirty percent reported disclosing their ART status to a friend, with 80% of females telling a female friend and 64% of males telling a male friend. Twenty-five percent described missing one or more doses of ART in the period between initiation and the six month study visit i.e., were non-adherent.

There were no major differences in the side-effects reported at six months between different first line ART regimens so we present Figure 1 detailing the side effects reported overall, across the different ART regimens taken (Figure 1).

In the six months after ART initiation, 60% reported having taken a nutritional supplement, six percent had taken herbal medication and two percent had taken anti-AIDS muti (a traditional African medication believed to boost the immune system) (Babb et al., 2007). By the six month visit, five percent had sought care from a traditional healer and 47% had visited a healthcare provider for a health condition other than HIV.

Univariably, the odds of non-adherence were significantly higher for those with the greatest level of perceived stigma compared to those with less perceived stigma and varied with levels of time spent with friends. However, neither stigma nor time spent with friends remained significant in the final multivariable model. There was no significant association between non-adherence and disclosing HIV status or disclosing ART initiation to either friends, family or partner, or any of the remaining variables measuring social support (Table 3).

**Table 3.** Final multivariable logistic regression for non-adherence in the last six months.

Variable *n * = 321	N (% non-adherent)	Unadjusted Odds Ratio (CI)^a^^,^b^^	Adjusted Odds Ratio (CI)	Likelihood Ratio Test *P* Value
Visited Public Healthcare service for condition other than HIV:				
No	169 (20)	1	1	0.01
Yes	152 (30)	1.79 (1.07–3.00)	1.98 (1.14–3.43)	
ARV Knowledge at enrolment:				
100% correct answers	157 (18)	1	1	<0.001
< 100% correct answers	164 (30)	1.94 (1.15–3.26)	2.10 (1.21–3.66)	
CD4 count at enrolment				
0–99 cells/ µl	103 (17)	1	1	0.01
100–199 cells/ µl	183 (27)	1.78 (0.97–3.25)	2.50 (1.30–4.81)	
≥200 cells/ µl	35 (31)	2.16 (0.90–5.20)	2.85 (1.10–7.40)	
Symptoms of Rash/Itching:				
No	239 (21)	1	1	<0.001
Yes	82 (36)	2.07 (1.19–3.62)	2.48 (1.37–4.52)	
Tested Positive <1 year before the study				
No	109 (18)	1	1	0.02
Yes	212 (28)	1.72 (0.97–3.00)	2.00 (1.10–3.70)	
Age:				
<48 years	273 (27)	1	1	<0.001
≥48 years	48 (10)	0.68 (0.50–0.94)	0.65 (0.46–0.90)	
Stigma				
Lesser perceived stigma	229 (21)	1		0.04
Greatest perceived stigma	92 (33)	1.78 (1.04–3.04)		
Disclosed HIV status at enrolment:				
Disclosed to family	241 (26)	1.00		0.66
Disclosed to none	35 (20)	0.71 (0.30–1.70)		
Disclosed to friends	3 (33)	1.41 (0.13–15.85)		
Disclosed to both	42 (19)	0.66 (0.30–1.51)		
ART Disclosure				
Not Disclosed	24 (29)	1		0.43
Disclosed to FAM	200 (22)	0.66 (0.26–1.70)		
Disclosed to FRI	4 (25)	0.81 (0.07–9.18)		
Disclosed to both	93 (30)	1.05 (0.39–0.99)		
ART Disclosur				
Rely on Family/Friends				
A Lot	244 (25)	1		0.87
A Little	66 (24)	0.96 (0.51–1.81)		
Not at All	11 (18)	0.53 (0.14–3.17)		
Open with Family/Friends				
A lot	122 (23)	1		0.85
A little	119 (25)	1.13 (0.62–2.04)		
Not at All	80 (26)	1.29 (0.62–2.30)		
Time with Family:				
Every Day	180 (30)	1		0.13
Several days/week	13 (15)	0.44 (0.15–1.36)		
At least once a fortnight	25 (16)	0.37 (0.11–1.30)		
Once a month	81 (20)	0.57 (0.30–1.08)		
Less than once a month	22(14)	0.42 (0.09–1.98)		
Time with Friends				
Less than once a month/once a month		1.00		0.02
At least once a fortnight		3.97 (1.46–10.77)		
Several days a week/Every day		1.46 (0,85–2.49)		

Note: *N* = 321.^a^^,^^b^

^a^Other variables that were significant only in univariable models (data not shown): having visited a traditional healer in the last six months, having more gender equitable norms, having ever drunk alcohol were associated with greater odds of non-adherence.

^b^In univariable analysis, we also considered HIV optimism, ART drug regime, ART disclosure to partner and gender but these were not significant.

Table 3 presents all variables significant in the final multivariable model, and shows that the odds of non-adherence were significantly higher for: those who had less than complete ART knowledge (aOR 2.10 CI: 1.21–3.66); who visited a healthcare provider for a condition other than HIV (aOR: 1.98, CI: 1.14–3.43); had higher CD4 counts (CD4 100-199: aOR 2.50 CI: 1.30–4.81; CD4 ≥ 200: aOR 2.85, CI: 1.10–7.40 compared to CD4 < 100); tested positive in the year before enrolment (aOR 2.00, CI: 1.10–3.72) and experienced rash/itching as a side-effect of ART (aOR 2.48,CI: 1.37–4.52). The odds of non-adherence were significantly lower for those ≥48 years (aOR 0.65, CI: 0.46–0.90).

## Recent non-adherence

Nine percent described missing one or more doses of ART in the month prior to the six month interview. We found no association between this recent non-adherence outcome and social support, HIV, ART disclosure or perceived stigma.

Applying the final multivariable model in the previous section to this outcome, we found that only ART knowledge (aOR 2.39 CI 1.03–5.55, *p* = 0.04) was significant and had a similar odds ratio estimate compared to our primary non-adherence estimate. The odds ratio estimates for age, testing positive and visiting a healthcare provider for a condition other than HIV were substantially attenuated indicating that they were not associated with recent non-adherence. The other variables remained similar in odds ratio estimate to the “non-adherence” model; however, were not quite significant which suggests this change was due to lower power. Building a final multivariable model for the recent non-adherence outcome, we found that having taken a nutritional supplement in the last six months (aOR 3.08 CI 1.19–7.94 *p* = 0.01) and using herbal medicine in the last six months (aOR 3.73 CI 1.18–11.78 *p* = 0.04) were associated with recent non-adherence.

### Discussion

We found no association between social support, stigma or disclosure of HIV and ART status and non-adherence. Although we found that a greater level of perceived stigma and spending time with friends were significant univariably, they were not significant in adjusted models. Whilst our finding of no association between social support and adherence is consistent with Ncama et al. (2008), there may be other explanatory factors (Ncama et al., 2008). Firstly, in our study, all participants were engaged with HIV care. A past study suggests that those within HIV care are more likely to have stable partnerships and hence greater social support (Conroy et al., 2017); therefore we think our sample population may have had higher overall social support. The quality of relationship with a primary partner and the presence of trust has been highlighted by past research as a central aspect of social support in enhancing ART adherence (Conroy et al., 2017; Kiwuwa-Muyingo et al., 2012). However, we found no association between disclosure to a partner and non-adherence. Secondly, past studies indicate that depression and lack of belief in own self-efficacy are associated with both low social support and increased non-adherence in HIV-positive adults in SSA (Conroy et al., 2017; Hunter-Adams et al., 2017; Kekwaletswe, Jordaan, Nkosi, & Morojele, 2016; Ncama et al., 2008). Our study did not measure these factors, so it is possible that residual confounding by these factors contributed to the difference between our results and previous research Finally, the majority of research in this area has collected qualitative data, which may better reflect relationship dynamics and individualised support networks provided by friends and family than the quantitative measures used in our analyses (Conroy et al., 2017; Hunter-Adams et al., 2017).

Twenty-five percent reported non-adherence in the first six months, which is comparable to other studies reporting 21–38%, in SA at a similar time (Bhat et al., 2010; Bhengu et al., 2011). A far lower proportion were non-adherent in the month before the six month interview. This change is consistent with findings from other studies in SSA which showed that ART adherence improved over time in HIV-positive adults, particularly in the three months after initiation (Bijker et al., 2017; Kiwuwa-Muyingo et al., 2012; Maqutu, Zewotir, North, Naidoo, & Grobler, 2011). The DART trial authors postulated that a learning effect from ART counselling and support may be partly responsible. They emphasised the need for better assessment of adherence built into routine clinics (Demessie, Mekonnen, Amogne, & Shibeshi, 2014; Kiwuwa-Muyingo et al., 2012; Loeliger et al., 2016). Our finding that incomplete ART knowledge at enrolment was significantly associated with higher odds of non-adherence supports this hypothesis. Whilst these studies propose that patients need accurate ART knowledge, one based in SA suggested that ART counselling needs to continue beyond ART initiation and that longitudinal support needs to be available to patients from the point of diagnosis to facilitate early adherence (Demessie et al., 2014; Loeliger et al., 2016). Therefore, we suggest early reinforcement of ART education, alongside routine adherence monitoring (Demessie et al., 2014; Kiwuwa-Muyingo et al., 2012; Loeliger et al., 2016). Our findings that a higher CD4 count at initiation and a recent first positive HIV-test were associated with increased non-adherence are important (Department of Health, 2016). They suggest that the awareness of heightened disease severity and illness experience are associated with motivation to adhere (Gao, Nau, Rosenbluth, Scott, & Woodward, 2000; Kiwuwa-Muyingo et al., 2012; Singh et al., 1996). ART counselling that reinforces the importance of taking medication even when feeling well may enhance adherence.

Visiting a healthcare provider for a condition other than HIV was associated with increased odds of non-adherence in the six months since ART initiation. We also found that taking a nutritional supplement or herbal medication were associated with recent non-adherence. These behaviours may be indicative of multimorbidity or care-seeking for ART side effects, which have been found by previous studies to be associated with higher odds of non-adherence, although we do not have additional data to confirm this in our analysis (Demessie et al., 2014; Nachega, Hsu, Uthman, Spinewine, & Pham, 2012). As the perception of HIV shifts towards that of a chronic condition and the prevalence of non-communicable disease rises in SA, understanding ART adherence within the context of multimorbidity will become vital (Mayosi et al., 2009; Mendenhall & Norris, 2015). We considered the number of reported side-effects as a possible confounder and found it was not significant. However, the presence of rash/itching was associated with non-adherence (see Figure 1 for details of side-effect patterns reported). Patients’ incomplete understanding of the potential side-effects of ART may limit their ability to seek help either socially or from healthcare workers (Ruud, Srinivas, & Toverud, 2012). ART counselling which incorporates teaching on side-effects may be beneficial.

The strength of this study is the detailed data on HIV disclosure patterns, stigma, social support and the prevalence of individual side-effects. There were limitations to this study. ART adherence assessment relied on self-report, and it is possible that some participants feared disclosing non-adherence (Simoni, Kurth, et al., 2006). Our results may also underestimate the true level of non-adherence because those who had died or been lost to follow-up may have been more likely to be non-adherent had they initiated ART but were not included in our analyses. Additionally, these analyses examined ART adherence in the first six months which may differ from longer term adherence to ART and thus we caution against extrapolating the results beyond the focus of this paper. Reports of variables such as alcohol use and health seeking behaviour at six months represented the interval between initiation and the six months study visit thus the temporality of these factors in relation to the timing of the non-adherence is less clear (Kiwuwa-Muyingo et al., 2012). Since this study was conducted, guidelines regarding ART initiation have changed, and people living with HIV are encouraged to initiate ART upon diagnosis. However, despite expansion of the ART programme, studies suggest that linkage of those living with HIV to ART treatment is sub-optimal (Haber et al., 2017; Iwuji et al., 2016). A recent study states that from 2005 to 2012 the proportion of those entering HIV care with a CD4 count <200 cells decreased from 46.6% to 32.9% and then plateaued around 32.9%–34.8% between 2012 and 2016 (Carmona et al., 2018). This data indicates that despite improvements in HIV guidelines, ART access for the current patient population has not improved, so many will have CD4 counts at treatment initiation comparable to those of our study cohort.

In our setting, social support, HIV disclosure and perceived stigma were not associated factors. Our findings suggest it is important that rollout of immediate ART post-diagnosis includes a programme of reinforcement of ART knowledge and routine monitoring of adherence incorporated into clinic appointments to support early ART adherence.

## Data Availability

The analytical dataset for this study is available through request to the Africa Health Research Institute data repository (https://data.africacentre.ac.za).
